# Modulation of Photorespiratory Enzymes by Oxidative and Photo-Oxidative Stress Induced by Menadione in Leaves of Pea (*Pisum sativum*)

**DOI:** 10.3390/plants10050987

**Published:** 2021-05-15

**Authors:** Ramesh B. Bapatla, Deepak Saini, Vetcha Aswani, Pidakala Rajsheel, Bobba Sunil, Stefan Timm, Agepati S. Raghavendra

**Affiliations:** 1 Department of Plant Sciences, School of Life Sciences, University of Hyderabad, Hyderabad 500046, India; rameshbptl@gmail.com (R.B.B.); sainideepak284@gmail.com (D.S.); ashu.6489@gmail.com (V.A.); rajsheelplantbiotech@gmail.com (P.R.); b.sunil@hotmail.com (B.S.); 2 Plant Physiology Department, University of Rostock, Albert-Einstein-Straße 3, D-18051 Rostock, Germany; stefan.timm@uni-rostock.de

**Keywords:** cellular compartments, chloroplasts, high light, menadione, mitochondria, oxidative stress, peroxisomes, photorespiration

## Abstract

Photorespiration, an essential component of plant metabolism, is concerted across four subcellular compartments, namely, chloroplast, peroxisome, mitochondrion, and the cytoplasm. It is unclear how the pathway located in different subcellular compartments respond to stress occurring exclusively in one of those. We attempted to assess the inter-organelle interaction during the photorespiratory pathway. For that purpose, we induced oxidative stress by menadione (MD) in mitochondria and photo-oxidative stress (high light) in chloroplasts. Subsequently, we examined the changes in selected photorespiratory enzymes, known to be located in other subcellular compartments. The presence of MD upregulated the transcript and protein levels of five chosen photorespiratory enzymes in both normal and high light. Peroxisomal glycolate oxidase and catalase activities increased by 50% and 25%, respectively, while chloroplastic glycerate kinase and phosphoglycolate phosphatase increased by ~30%. The effect of MD was maximum in high light, indicating photo-oxidative stress was an influential factor to regulate photorespiration. Oxidative stress created in mitochondria caused a coordinative upregulation of photorespiration in other organelles. We provided evidence that reactive oxygen species are important signals for inter-organelle communication during photorespiration. Thus, MD can be a valuable tool to modulate the redox state in plant cells to study the metabolic consequences across membranes.

## 1. Background

Photosynthesis in higher plants is affected by abiotic stress such as oxidative conditions and high light (HL) intensities. Prolonged exposure to such stress can damage the photosynthetic apparatus, particularly PSII, resulting in photoinhibition [1,2,3]. Nevertheless, plants try to protect photosynthesis against photoinhibition by operating different compartments of their cells [4]. Interactions between chloroplasts and mitochondria through peroxisomes and cytosol are essential for optimizing photosynthesis [4,5,6,7]. In an earlier report, Saradadevi and Raghavendra [8] demonstrated that mitochondrial oxidative electron transport and phosphorylation could protect photosynthesis against photodamage in pea mesophyll protoplasts. In addition to dark mitochondrial respiration, photorespiration is now acknowledged to be a protective mechanism against photoinhibition, as indicated by the classic work on photorespiratory mutants [9,10,11]. Readers interested in photorespiratory metabolism can refer to several reviews, which appeared periodically [12,13,14,15,16]. In addition, photorespiration is a classic example of the compartmentation in different cellular organelles [13,16]. Interestingly, the coordination of photorespiratory metabolism between mitochondria and peroxisomes was also demonstrated in vitro reconstructed systems [17].

The biochemical link between photorespiration, photosynthesis, and other assimilatory pathways is recognized and is currently of great attention in plant biology [18,19,20]. Despite being a major source of reactive oxygen species (ROS) in peroxisomes, photorespiration is a part of plant stress responses. In different abiotic stress conditions, such as drought, HL, or cold, photorespiration can minimize the ROS levels by preventing accumulation, particularly during oxidative stress [4,11,21]. Photorespiration contributes to the protection of PSII from oxidative stress and PSI by optimizing its redox state [22]. Under CO_2_ limiting conditions or HL, photorespiration can be a sink for excess reductants and maintain the redox state by removing ATP and NADPH [23,24]. When tobacco plants were exposed to HL, increased photorespiration plays a vital role in energy balancing along with activation of the water-water cycle [25].

Another important intersection of photorespiration with other cellular metabolism is nitrogen assimilation and utilization, mainly because of the refixation of ammonia generated by photorespiration in the mitochondria [13,15]. In addition, proper photorespiratory flux is needed for nitrate assimilation, as demonstrated in various species [12,26,27]. Moreover, plastidic glutamine synthetase (GS2) turned out to be a crucial component as well as studies on mutants categorically established the indispensable role of GS2 in photorespiration [9,28,29]. However, even cytosolic GS1 might play a role in the upregulation of photorespiration under certain situations [30].

We pointed at the possible signals between chloroplasts, mitochondria, and cytoplasm to optimize photosynthetic carbon metabolism [4,7]. The ability of ROS as a mobile signal to modulate the metabolism at subcellular levels is known [31,32]. Recently, we observed that treatment of pea leaf discs with either menadione (MD; targets initially mitochondria) or paraquat (targets chloroplasts) increased cellular ROS, including superoxide and H_2_O_2_ [33]. This was followed by the upregulation of antioxidant enzymes and a rise in proline levels, indicating that an increase in ROS can be a signal to modulate the metabolism in both chloroplast and mitochondria.

However, there are still gaps in our knowledge on the operation and regulation of photorespiration. Some of these are (i) exact molecular/biochemical signals that trigger the onset of photorespiration; (ii) mobility of such signal between cellular compartments; (iii) mechanisms and extents of metabolite fluxes. The present article provides some answers and demonstrates that ROS (both superoxide and H_2_O_2_) can be a signal that traverses across the organelles and modulate the critical enzymes of photorespiration.

Our primary goal was to trigger ROS production in mitochondria by MD and examine if photorespiratory enzymes in other cellular compartments respond. In leaves, a significant increase in ROS levels occurs on exposure to high light and ensuring photoinhibition [24,31]. Under high light, the stress is initiated in chloroplasts, while the primary target of menadione is mitochondria. Thus, we have two different targets for triggering stress. Irrespective of the target being chloroplasts or mitochondria, the key enzymes of photorespiration in other compartments, particularly peroxisomes and chloroplasts, were modulated.

We are also aware that mitochondrial metabolism offers protection against photoinhibition. We have therefore evaluated the effects of MD in dark, normal, and high light. Severe increases in ROS levels (superoxide and H_2_O_2_), antioxidants, and antioxidant enzymes confirmed the imposition of oxidative stress by MD. There were marked increases in the transcripts, proteins, and activities of typical photorespiratory enzymes, known to be located in either peroxisomes (such as glycolate oxidase (GO), catalase (CAT), hydroxypyruvate reductase (HRP)) or chloroplasts (glycerate kinase (GK) and 2-phosphoglycolate phosphatase (PGLP)). When leaf samples were treated with a scavenger of H_2_O_2_ (catalase) or superoxide (Tiron), the increases in the activities of GO and GK in response to MD were dampened, suggesting both superoxide and H_2_O_2_ (two forms of ROS) were largely responsible for the co-responses.

## 2. Results

Both superoxide and H_2_O_2_ accumulated in pea leaf discs with increased MD concentrations (Supplementary Figure S1) and over time (Supplementary Figure S2). Based on the kinetics of ROS (superoxide and H_2_O_2_) accumulation, we chose a combination of 10 µM MD and 3 h of exposure time for subsequent experiments.

### 2.1. Changes in Levels of ROS and Antioxidants

**Figure 1:**
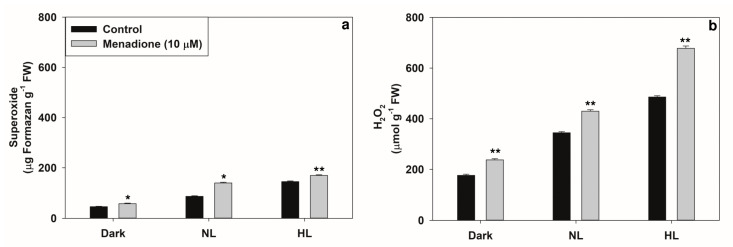
Leaf discs were treated with menadione (MD 10 µM) and incubated under dark, normal (NL, 300 µmol m^−2^ s^−1^) or high light (HL, 1200 µmol m^−2^ s^−1^) light for 3 h and quantification of generated superoxide and H_2_O_2_. Another set had no MD, but were kept in dark, NL or HL. (**a**) Formazan, precipitate formed from the reduction of NBT by superoxide. (**b**) Levels of H_2_O_2_ detected by DAB-staining. Each data point is the average of three replicates and error bars represent ±SE. Asterisks indicate the significance of MD effect compared to the respective control. * *p* < 0.05; ** *p* < 0.001.

**Figure 2:**
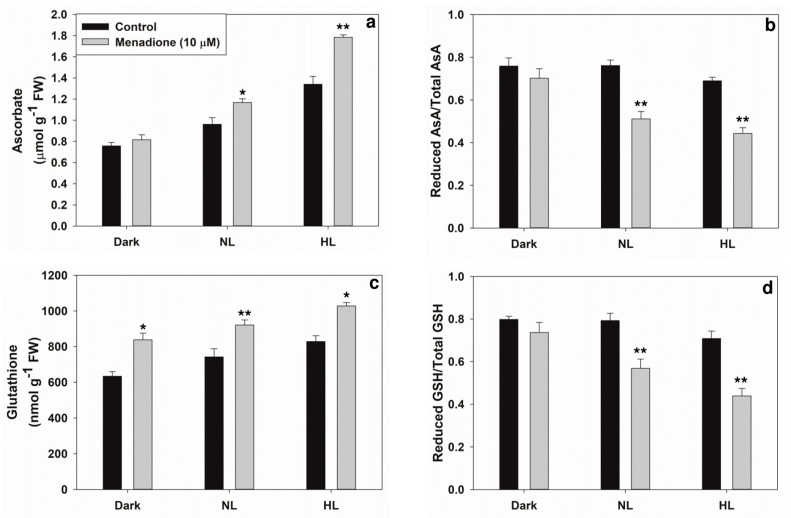
The levels of antioxidants: ascorbate (**a**) and glutathione (**c**) and their redox ratios (**b**,**d**) on treatment with light and/or 10 µM MD. Each data point is the average of three replicates, and error bars represent ±SE. Asterisks indicate the significance of MD effect compared to the respective control. * *p* < 0.05; ** *p* < 0.001.

The patterns of ROS accumulation on exposure to MD under normal light (NL) or high light (HL) were ensured (Figure 1a,b). There was a significant increase in the ROS (both superoxide and H_2_O_2_) content under NL as well as in HL conditions on exposure to menadione. Superoxide content was increased by MD up to 60% above control, while H_2_O_2_ content increased by up to ~40% on exposure to MD. The levels of both ascorbate and glutathione significantly increased under NL and HL conditions in the presence of MD (Figure 2a,c). In contrast, the ratios of reduced to total ascorbate or glutathione were decreased in the presence of MD (Figure 2b,d).

### 2.2. Changes in Transcripts and Activities of Key Antioxidant Enzymes

**Figure 3:**
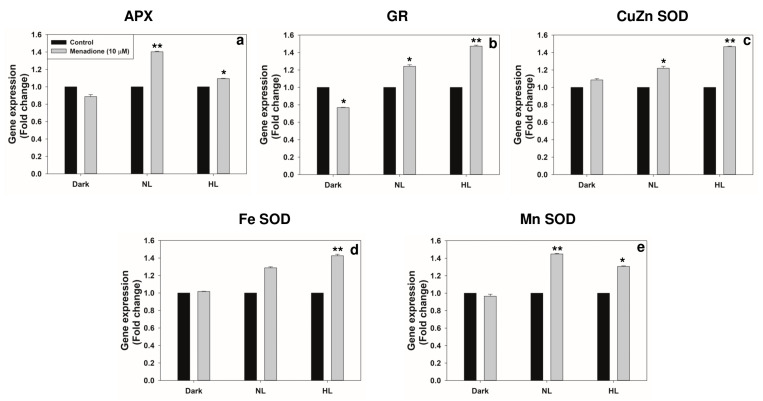
The transcript levels of antioxidant enzymes: ascorbate peroxidase; APX (**a**); glutathione reductase; GR (**b**); copper zinc superoxide dismutase; CuZnSOD (**c**); iron superoxide dismutase; FeSOD (**d**); manganese superoxide dismutase; MnSOD (**e**) on exposure to light in the absence or presence of MD. The expression of genes was represented in fold change after normalization with Actin 2. Each data point is the average of three replicates, and error bars represent ±SE. Asterisks indicate the significance of MD effect compared to the respective control. * *p* < 0.05; ** *p* < 0.001.

**Figure 4:**
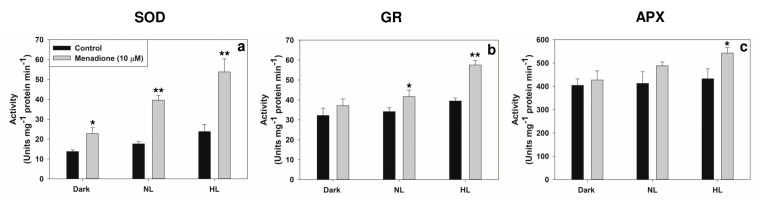
The activities of typical antioxidant enzymes superoxide dismutase, SOD (**a**), glutathione reductase, GR (**b**), ascorbate peroxidase, APX (**c**) in leaf extracts on treatment with menadione in normal (NL) or high light (HL). Each data point is the average of three replicates, and error bars represent ±SE. Asterisks indicate the significance of MD effect compared to the respective control. * *p* < 0.05; ** *p* < 0.001.

The transcripts of superoxide dismutase (Cu/Zn-SOD and Fe-SOD) and glutathione reductase (GR) were upregulated under HL by MD. Only minor changes occurred in the amounts of ascorbate peroxidase (APX) or Mn-SOD (Figure 3). The activities of antioxidant enzymes SOD and GR increased significantly in MD-treated samples, under both NL and HL (Figure 4a,b). In contrast, the APX activity increased only under HL with MD. Even in the dark, a slight increase in the SOD activity could be seen, but there was not much effect on APX or GR (Figure 4b,c).

### 2.3. Modulation of Photorespiratory Enzymes in Peroxisomes and Chloroplasts by MD and/or High Light

**Figure 5:**
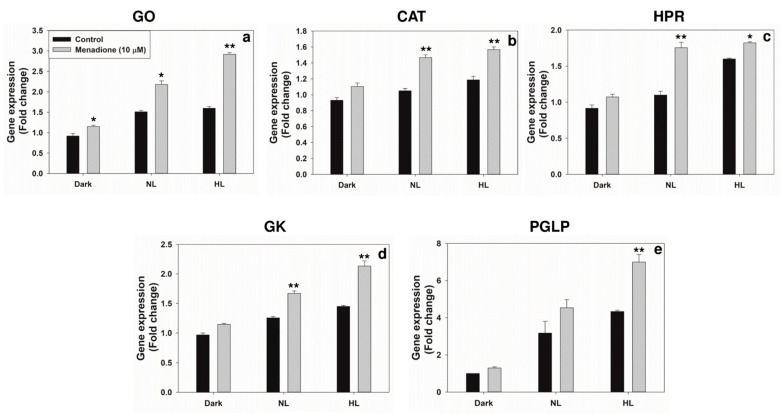
The transcript levels of glycolate oxidase (GO) (**a**), catalase (CAT) (**b**), hydroxypyruvate reductase (HPR) (**c**), glycerate kinase (GK), (**d**), and phosphoglycolate phosphatase (PGLP) (**e**), in extracts from leaf discs on exposure to MD and/or high light (HL, 1200 µmol m^−2^ s^−1^). The expression of genes was represented as fold-change after normalization with actin 2. Each data point is the average of three replicates, and error bars represent ±SE. Asterisks indicate the significance of MD effect compared to the respective control. * *p* < 0.05; ** *p* < 0.001.

**Figure 6:**
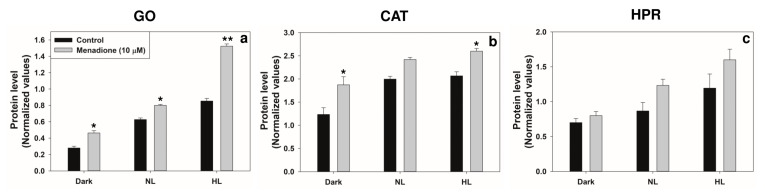
The levels of enzyme proteins: glycolate oxidase (GO) (**a**), catalase (CAT) (**b**), and hydroxypyruvate reductase (HPR) (**c**), in extracts from leaf discs on treatment with MD either normal or high light (1200 µmol m^−2^ s^−1^). The ratios of each protein (such as GO, CAT, and HPR) to Rubisco large subunits (loading control) were calculated based on ImageJ software. Each data point is the average of three replicates, and error bars represent ±SE. Asterisks indicate the significance of MD effect compared to the respective control. * *p* < 0.05; ** *p* < 0.001.

We examined the transcripts and protein levels of GO, CAT, HPR, GK, and PGLP, in the leaf extracts. When pea leaf discs were treated with MD, the transcripts of enzymes, such as GO, CAT, and HPR increased, particularly in HL (Figure 5). Similarly, the Western blots revealed that their protein levels also increased in HL and MD treatments (Figure 6).

**Figure 7:**
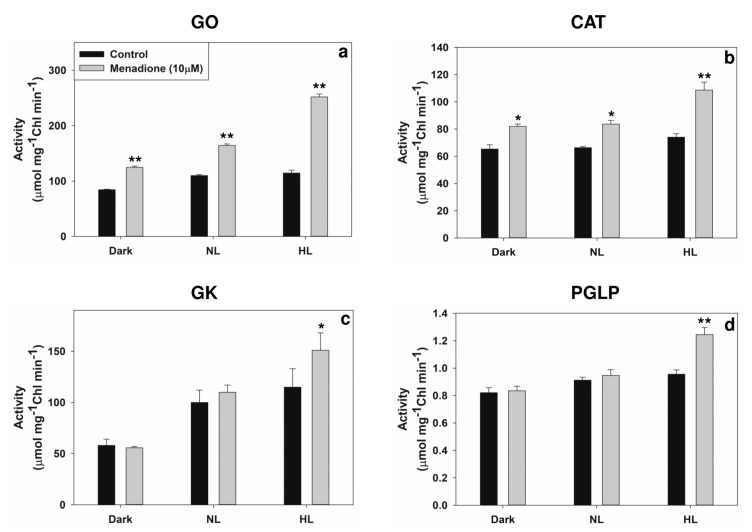
The activities of key photorespiratory enzymes: glycolate oxidase (GO) (**a**), and catalase (CAT) (**b**), glycerate kinase (GK) (**c**), and phosphoglycolate phosphatase (PGLP) (**d**) in leaf extracts from leaf discs on treatment with MD and/or high light. Each data point is the average of three replicates, and error bars represent ±SE. Asterisks indicate the significance of MD effect compared to the respective control. * *p* < 0.05; ** *p* < 0.001.

**Figure 8:**
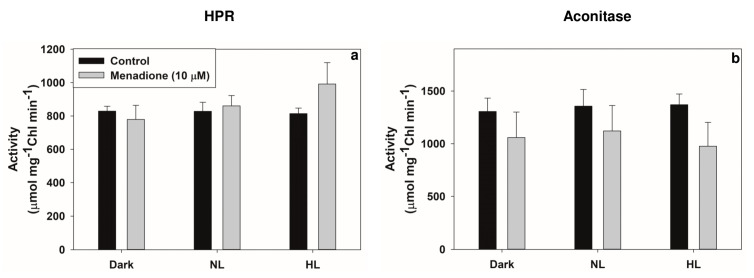
The activities of two photorespiratory enzymes, peroxisomal hydroxypyruvate reductase, HPR (**a**) and mitochondrial aconitase (**b**) in extracts from MD (10 µM) treated leaf samples.

When exposed to MD and light, the activities of two peroxisomal enzymes, GO, and CAT increased (Figure 7a,b). The levels of glycolate and glyoxylate increased in MD-treated samples, particularly in HL. However, the ratio of glycolate/glyoxylate decreased in MD-treated samples. In contrast, there was only a slight increase in the activity of another peroxisomal enzyme, HPR (Figure 8a), while the two chloroplastic enzymes, GK and PGLP, are significantly increased by ~30% on exposure to MD in HL (Figure 7c,d). In contrast to the photorespiratory enzymes, the activity of aconitase, a mitochondrial enzyme, considered an indicator of oxidative stress, was suppressed in MD-treated samples compared to control (Figure 8b).

### 2.4. Reversal of Stress-Induced Responses by Superoxide or H_2_O_2_ Scavengers

**Figure 9:**
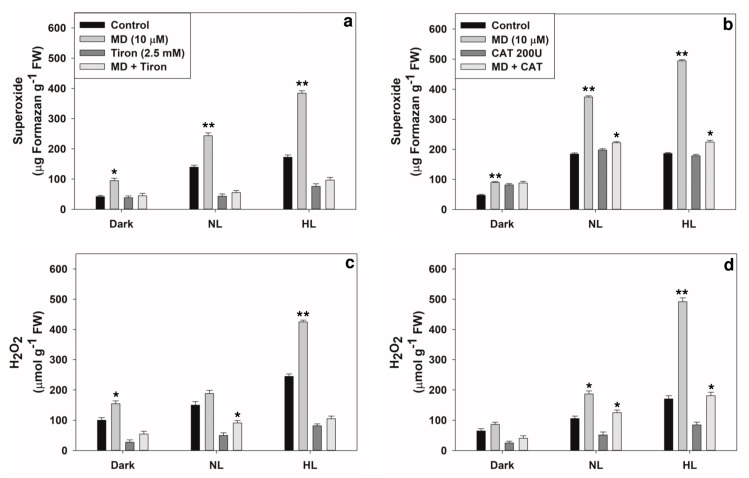
Scavenging ROS, superoxide or H_2_O_2_ by Tiron (2.5 mM) or catalase (200 U), respectively, in pea leaf discs after treatment with or without MD. The levels of superoxide (**a**,**b**) and H_2_O_2_ (**c**,**d**) were determined using NBT and DAB, respectively. * *p* < 0.05; ** *p* < 0.001.

**Figure 10:**
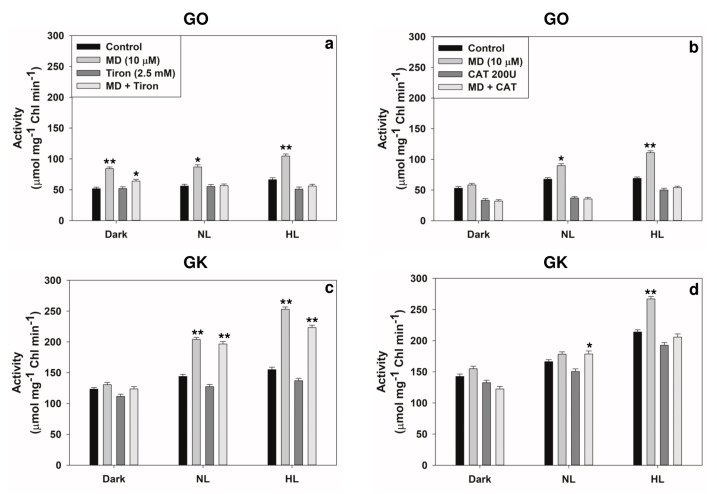
The consequence of scavenging superoxide (by Tiron) or H_2_O_2_ by (catalase) on the activities of two key photorespiratory enzymes: glycolate oxidase (GO) (**a**,**b**) and glycerate kinase (GK) (**c**,**d**) in extracts from the leaf discs. * *p* < 0.05; ** *p* < 0.001.

Next, we examined CAT and Tiron as scavengers of H_2_O_2_ and superoxide, respectively [34,35]. CAT was able to scavenge H_2_O_2_ more efficiently than superoxide. However, the scavenging by Tiron of superoxide and H_2_O_2_ were similar (Figure 9). When present, both CAT and Tiron restricted the extent of MD-induced increase in the activity of two photorespiratory enzymes, namely GO and GK (Figure 10).

## 3. Discussion

Plants especially need to cope with fluctuating light intensity and a combination of abiotic and biotic stresses [36], as elevated levels of ROS under stress can cause harmful effects on plant metabolism [3]. However, photo-oxidative stress was observed to cause more damage than oxidative stress [33]. Our results too emphasized that oxidative stress induced by MD was significant and quite pronounced under HL. The present report demonstrates that redox perturbations by MD in mitochondria can upregulate photorespiratory enzymes in other cellular compartments, namely peroxisomes and chloroplasts. Although MD was used to study the responses of plant systems to stress earlier, e.g., 60 to 400 µM in the case of *Arabidopsis* [37,38,39,40,41] and up to 0.2 mM in pea, *Pisum sativum* [42,43]. We used a much lower concentration (10 µM) to avoid unspecific effects that are seen at high concentrations of inhibitors.

We used high light and oxidative stress (by MD), as such combination occurs in nature [44,45,46]. The amplified effects of oxidative stress (due to drought or other conditions) under high light conditions are reflected in the responses of photosynthesis and photoinhibition [47,48]. Our work demonstrated the importance of mitochondrial metabolism in protecting photosynthesis against photoinhibition. The inhibition of photosynthesis under supra-optimal light gets aggravated when mitochondrial electron transport was blocked [4,8]. The marked modulation of photorespiratory enzymes by high light, amplified further by MD-treatment, is therefore not surprising.

### 3.1. ROS as Signals to Modulate the Metabolism in Multiple Compartments of Plant Cells

The process of photorespiration is an essential component of abiotic stress responses and can help adapt to a multitude of environmental parameters [11,49,50,51]. Further, the benefits of photorespiration were proposed to be complemented by chloroplastic cyclic electron transport and mitochondrial alternative oxidase [21]. Our results add another dimension to emphasize the signaling role of ROS coordinating the responses in different compartments of mitochondria, chloroplasts, and peroxisomes. The modulation of ROS is the basis of plant adaptation under such a combination of stresses [32,52].

Under HL stress, a significant increase in the levels of superoxide and H_2_O_2_ occurred on the treatment of pea leaf discs with MD (Figure 1), similar to earlier reports [33,37,41,53,54]. ROS are essential signals to acclimatize plants to different stresses. Chloroplastic superoxide is known to be the major source of ROS in leaves. When exposed to HL, chloroplasts produce considerable amounts of superoxide from excited chlorophyll molecules and the disruption of the balance between PSI and PSII reaction centers (31). MD is a redox-active quinone analog that causes oxidative stress by forming superoxide radicals, primarily in mitochondria (38). MD can also target sulfhydryl groups and reduce GSH levels (39,41). Experiments with redox-sensitive GFP (m-roGFP2) revealed that MD created oxidative stress in mitochondria, and the stress later spread to other compartments [39]. MD can generate ROS in the dark, too, as indicated by the limited increase in ROS levels compared to the control samples. This is not surprising, as Mor et al. [55] reported that an increase in ROS when fluorescent (flu)-like mutants of *Arabidopsis thaliana* were exposed to abiotic (Rose Bengal) or biotic stress, even in the dark.

Methyl viologen-, MD-, and H_2_O_2_-induced cell death in Arabidopsis leaves underlined mitochondria and cytoplasm interactions [56]. In addition to being a source of ROS, MD can help plants to acclimatize to low temperatures. The exposure to HL (abiotic stress) and menadione (an oxidant) triggering photo-oxidative stress can lead to priming of plants to future exposures to abiotic stress. The primed state of plants has been related to efficient activation of defense responses and also enhanced resistance to recurring challenges of stress [57]. Thus, menadione can act as a potent priming agent to induce tolerance against abiotic stresses [58,59].

In our experiments, when mitochondria were targeted with MD, the photorespiratory components in peroxisomes and chloroplasts responded (Figure 7a–d). We also attempted to prove whether MD-induced changes in the activities of photorespiratory enzymes can be directly related to ROS increases. The modulation by MD of photorespiratory enzymes was dampened when the scavengers of superoxide (Tiron) and H_2_O_2_ (catalase) were present in the incubation medium (Figure 10). Thus, the changes were obviously due to the overall accumulation of superoxide and H_2_O_2_ in leaves (Figure 1).

### 3.2. Modulation of Photorespiratory Enzymes Located in Peroxisomes and Chloroplasts by MD and High Light

Photorespiration is an important factor that can protect photosynthesis against oxidative damage under HL and other abiotic stress conditions, including drought or salinity [21]. An increase in the transcripts and enzyme activities of key photorespiratory enzymes such as GO or CAT under HL or osmotic stress was noticed before [11]. However, observations on collective responses of enzymes distributed among several subcellular compartments are rare.

Aconitase, another enzyme located in mitochondria, provided an interesting comparison. The activity of aconitase was decreased on exposure to MD and HL (Figure 8b). Aconitase is known to be sensitive to oxidative stress [39]. In an earlier study, with heterotrophically grown Arabidopsis cells, exposure to MD inhibited the aconitase activity [53]. For example, pretreatment with MD, caused oxidative stress leading to the degradation of mitochondrial proteins, inhibition of TCA cycle enzyme metabolism, and changes in the NADPH pools [37,41].

Despite the primary disturbance in mitochondria, photorespiratory enzymes located in peroxisomes and chloroplasts responded to MD treatments. For example, GO/CAT (peroxisomal) and GK/PGLP (chloroplastic) were upregulated in MD-treated leaf samples in HL (Figure 7a–d). In agreement, increases in GO and CAT activity were observed in other studies under HL or drought [60,61,62,63,64]. Similarly, the activities of chloroplastic GK and PGLP were upregulated on treatment with paraquat [65,66] or heavy metals [67], suggesting that photorespiration could contribute to the protection against over-reduction of photosynthetic components.

Arabidopsis mutants were used to study metabolism during the acclimation to short-term ambient CO_2_ conditions. These mutants exhibited an increase in pools of key metabolites such as glycolate, glycerate, glycine, and serine, indicating that the photorespiratory flux of metabolites was involved in protecting photosynthesis [68,69]. In our experiments, the upregulation of photorespiration was reflected in not only the transcriptional and translational levels but also the activity of enzymes (Figure 7a–d; Figure 8). Based on the enzyme activities and gene expression pattern, we suggest that the MD could be a valuable tool to modulate the cellular redox and, eventually, the photorespiratory components.

## 4. Conclusions and Future Perspectives

ROS produced in either chloroplasts (HL-stress) or mitochondria (oxidative stress by MD) could move across the cell, modulating enzymes in other organelles, including peroxisomes and chloroplasts. Our study emphasizes the mobility of ROS (both superoxide and H_2_O_2_) within the cell and elaborates the influence of ROS on the redox state in plant cells, with a focus on photorespiratory enzymes. It is evident that mitochondrial ROS production through MD can modulate the photorespiratory pathway within different subcellular compartments to adapt metabolic fluxes to environmental changes due to a collective upregulation of the pathway.

Our observations throw open several challenging areas for further study. The photorespiratory enzymes chosen by us are all related to carbon metabolism. It would be interesting to examine the effect of high light and MD on the enzymes of N-metabolism, which are also compartmentalized and coupled with photorespiration. The long-term consequences of upregulated photorespiration under photo-oxidative stress are of great interest. In biological systems, including plants, whenever the organisms are exposed to stress, they are primed to tolerate the recurring stress in the future. The upregulation of photorespiration under photo-oxidative stress may also offer such an advantage to the plants. It would be interesting to examine if plants pre-exposed to stress can tolerate oxidative stress at a later stage somewhat better.

## 5. Materials and Methods

### 5.1. Plant Material and Growth Conditions

Pea seeds (*Pisum sativum* L., cv. Arkel) from Durga Seeds (Chandigarh, India) were soaked overnight in water and sterilized with sodium hypochlorite (4%). The washed seeds were allowed to germinate on blotting paper for 2–3 days, and then the seedlings were transferred to pots containing manure and soil. The plants were grown in a greenhouse with average temperatures of 30 °C day/ 20 °C night.

Discs (approximately 0.25 cm^2^) were prepared from pea leaves with a sharp paper punch and kept in 5-cm-diameter Petri dishes containing the incubation medium (2 mM potassium phosphate buffer pH 6.5, 1 mM CaCl_2_, and 1 mM KCl with or without test compound, 10 µM MD. The discs were then kept for 3 h in either dark or normal (NL, 300 µmol m^−2^ s^−1^) or high light (HL, 1200 µmol m^−2^ s^−1^). At the end of each treatment, the leaves were frozen and stored in liquid N_2_. We used these frozen samples for all further analyses: transcripts, proteins, and enzyme assays.

### 5.2. Chemicals/Antibodies

Menadione, NBT, DAB, catalase and Tiron (4, 5-Dihydroxy-1, 3-benzenedisulfonic acid disodium salt) and Premix-BCIP/NBT solution were obtained from Sigma–Aldrich (St. Louis, MO, USA). Primary antibodies for GO, CAT, HPR, GR, and SOD (CuZn-SOD and Fe-SOD) and secondary antibody goat anti-rabbit IgG conjugated with alkaline phosphatase were from Agrisera AB (Vännäs, Sweden). All the chemicals were of analytical grade.

### 5.3. Staining and Quantification of ROS

ROS accumulation was monitored by staining for superoxide or H_2_O_2_ with nitroblue tetrazolium chloride (NBT) and 3,3′-diaminobenzidine (DAB), respectively, by Kwon et al. [35]. Leaf discs were infiltrated with NBT (1 mg/mL) and DAB (1 mg/mL) for 5 min by using a vacuum infiltrator and then kept for treatment. After respective treatment, leaf discs were soaked in a mixture of ethanol:lactic acid:glycerol in the ratio of 3:1:1 (*v*/*v*/*v*). The pigments were removed with methanol, and the cleared leaf discs were photographed. H_2_O_2_ was visualized as a reddish-brown color, while superoxide radicals were detected as blue color formazan.

For quantification of H_2_O_2_ and superoxide, DAB- and NBT-stained leaves were powdered in liquid nitrogen and homogenized in 0.2 M HClO_4_ and 2 M KOH-DMSO (1/1.6) (*v*/*v*) solution. Extracts were centrifuged at 10,000 rpm for 10 min at 4 °C. The absorbance of the supernatant was measured at 450 nm for H_2_O_2_ and 630 nm for superoxide. The exact levels were determined by using a standard curve prepared with known levels of H_2_O_2_ in 0.2 M HClO_4_-DAB or NBT in KOH-DMSO mix. This procedure of quantification was a slight modification of method by Kwon et al. [35].

### 5.4. Antioxidants and Antioxidant Enzymes

After respective treatments, approximately 100 mg of leaf discs were collected and stored in liquid nitrogen. Total ascorbate content was estimated according to Gillespie et al. [70]. Reduced and total ASA were calculated using the regression between the ASA standards and their blank corrected A_525nm_. Glutathione content was measured as described by Griffith et al. [71]. Total GSH was determined using GSH standard curve, and reduced GSH was determined by the difference between the total GSH and the GSSG.

The powdered leaf samples were homogenized in 50 mM phosphate buffer pH 7.0 containing 1 mM phenyl methane sulfonyl fluoride. The homogenate was centrifuged at 10,000× *g* for 10 min, and the supernatant was used for APX and GR assays. Protein was estimated by Lowry’s reagent [72].

Antioxidant enzymes were assayed as per established procedures, as follows: APX, amount of AsA oxidized at A_290nm_, Nakano and Asada [73]; and GR-NADPH oxidation at A_340nm_, Jiang and Zhang [74]. Superoxide dismutase (SOD), samples were homogenized in 50 mM phosphate buffer pH 7.8. The homogenate was centrifuged at 12,000× *g* for 10 min, and the supernatant was used for the assay. SOD-NBT reduction monitored at A_560nm_, Beyer and Fridovich [75].

### 5.5. Transcript and Protein Levels

The leaf discs (100 mg fresh weight) after respective treatment were ground in liquid nitrogen. RNA was extracted as per the procedure described by Chomczynski et al. [76]. cDNA synthesis and q-PCR analysis of transcripts (GO, CAT, HPR, GK, PGLP, APX, GR, Cu/Zn SOD, Fe SOD, and Mn SOD) were done as described by Ahn et al. [77]. Gene-specific primers were designed based on the published sequence (http://www.ncbi.nlm.nih.gov, accessed on 13 March 2019) and using ESTs available in the cool season food legume database (www.coolseasonfoodlegume.org, accessed on 13 March 2019) using Primer3 software [78]. The primers’ sequences for RT-PCR amplification of genes encoding for GO, CAT, HPR, GK, PGLP, APX, GR, Cu/Zn SOD, Fe SOD, and Mn SOD and the housekeeping gene, actin 2 (Supplementary Table S1). Actin 2 was used as an internal control. After electrophoresis, the band intensities were quantified using ImageJ software and normalized with reference to band intensities of actin 2.

The protein levels were monitored by Western blotting using Agrisera AB (Vännäs, Sweden) antibodies, following the manufacturer’s recommendation. The protein band intensities were quantified using ImageJ software and with reference to the large subunit of RubisCO.

### 5.6. Photorespiratory Enzyme Assays and Chlorophyll

Pea leaf discs were ground in a mortar and pestle using liquid nitrogen into a fine powder and homogenized in extraction buffer. The extraction of leaf discs and assays of photorespiratory enzymes were all as per the established procedures, as follows: GO–glyoxylate formation from glycolate, Yamaguchi and Nishimura [79]; CAT–H_2_O_2_ consumption monitored at A_240nm_, Patterson et al. [80]; NADH-HPR–NADH utilization in the presence of hydroxypyruvate, Timm et al. [81]; GK–monitoring PGA formation using pyruvate kinase-lactate dehydrogenase, Kleczkowski and Randall [82]; and PGLP release of Pi from PGLP, Somerville and Ogren [83]. The assay of aconitase was in accordance with the study by Lehmann et al. [39]. Chlorophyll concentration in leaf extracts was determined using 80% (*v*/*v*) acetone [84].

### 5.7. Effect of ROS-Scavengers

The role of ROS in the modulation of photorespiratory enzymes was assessed by using the scavengers of superoxide (Tiron) or H_2_O_2_ (catalase, Sigma Aldrich). Catalase is a well-known scavenger of H_2_O_2_, and Tiron can scavenge superoxide [34,35]. Leaf discs treated with MD in presence or absence of Tiron or CAT and kept in the dark, NL, or HL for 3 h. These were complemented with the assay of GO and GK, as described above.

### 5.8. Statistical Analysis

Results presented are the average of at least three independent experiments done on different days. The results were analyzed by ANOVA. Each data point is the average of three replicates, and error bars represented ±SE. Asterisks indicated the significance of the effect of MD, compared to the respective control. * *p* < 0.05; ** *p* < 0.001.

## Supplementary Materials

The following are available online at https://www.mdpi.com/article/10.3390/plants10050987/s1, Figure S1. Varying accumulation of ROS in leaf discs of pea with the concentration of MD. Figure S2. The levels of ROS in pea leaf discs after treatment with MD (10 μM) for varying periods. Table S1. Primers used in the study.
