# Supplementary material for: Modulation of Photorespiratory Enzymes by Oxidative and Photo-Oxidative Stress Induced by Menadione in Leaves of Pea (Pisum sativum)
Source: Plants (Basel). 2021 May 15;10(5):987. doi: 10.3390/plants10050987 (PMC8156035; doi:10.3390/plants10050987)
Supplement: Supplementary file 1 [file plants-10-00987-s001.zip › plants-1180327-supplementary.pdf]

## Supplementary Data

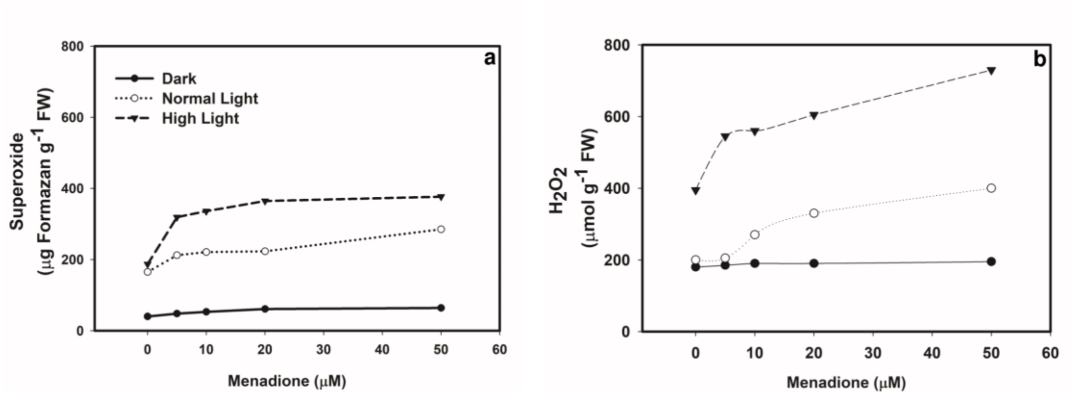


**Supplementary Figure S1.** Accumulation of ROS in leaf discs of pea with varying concentrations of MD (0 to 50 µM). **(a)** The superoxide levels were quantified as formazan formed after incubation with NBT, while the levels of $H_2O_2$ **(b)** were measured with DAB. The values given are the averages of two experiments done on different days.

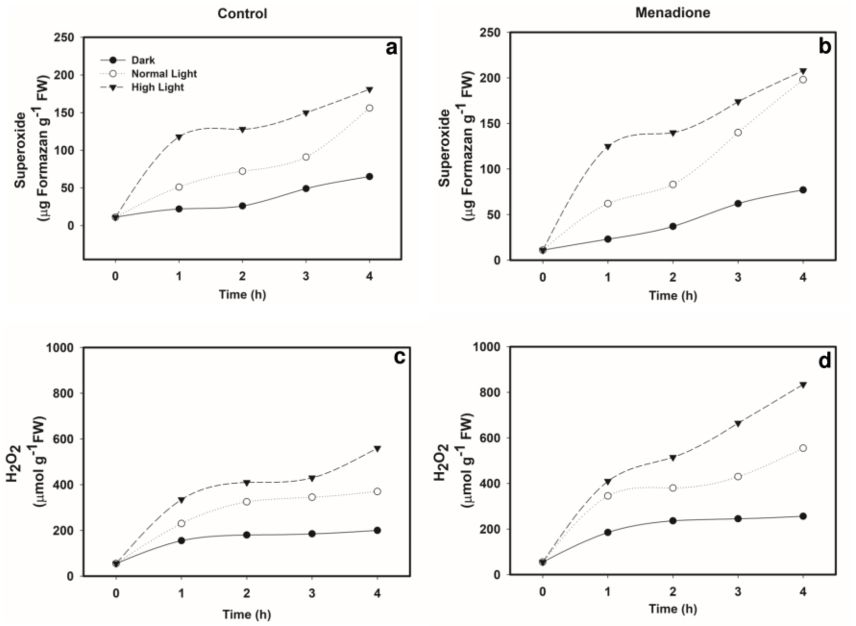


**Supplementary Figure S2.** ROS levels in pea leaf discs after treatment with MD (10 µM) for varying periods in dark, NL or HL intensities. Superoxide levels were quantified as formazan after incubating with NBT **(a and b)**, while the levels of $H_2O_2$ were measured by DAB staining **(c and d)**. The values given are the averages of two experiments done on different days.

*** * * * ***

**Supplementary Table S1.** Sequences of primers used in semi-quantitative RT-PCR. The complete genome of *Pisum sativum* is not yet available [1]. The primers were therefore designed on the basis of ESTs available in the cool-season food legume database (https://www.coolseasonfoodlegume.org/). When these primer sequences were run on BLAST, they showed 75% sequence identity with the enzymes in *Arabidopsis*.

| Gene | Primer sequence (5'-3') |
|---|---|
| *APX* | F: GGATCCTATGGGAAAATCATACCCAACTG<br>R: CTCGAGTCTTAGGCTTCAGCAAATCCAAG |
| *GR* | F: TTTTGCGAACACTGCTTTTG<br>R: AGCCTGAGGTGAAGACCAGA |
| *Cu/Zn SOD* | F: GAACAATGGTGAAGGCTGTG<br>R: GTGACCACCTTTCCCAAGAT |
| *Fe SOD* | F: GCACCACAGAGCTTATGTAG<br>R: GGAGTGGATGATGATGGTTC |
| *Mn SOD* | F: GGAGCAAGTTTGGTTCCATT<br>R: AAGGTTATTCGGCCAGATTG |
| *CAT* | F: CGAGGTATGACCAGGTTCGT<br>R: AGGGCATCAATCCATCTCTG |
| *GO* | F: GCTTCCCTGCGTCCGAGTCTT<br>R: GTGAGCAAGATCGACATG |
| *HPR* | F: CAGGTCCTC TAC TGC AGT<br>R: TTG GTG TTA TCG GTG CTGG |
| *GK* | F: ATCCCTATCTTCTGCTCC<br>R: GAC ATA CAC CGA TTT TCC |
| *PGLP* | F: GGCGGGGATGGT ATACTGGA<br>R: TTGATCCACACATGCCGC |
| *Actin 2* | F: AATGGTGAAGGCTGGATTTG<br>R: AGCAAGATCCAAACGAAGGA |

1. Kulaeva, O.A.; Zhernakov, A.I.; Afonin, A.M.; Boikov, S.S.; Sulima, A.S.; Tikhonovich, I.A.; Zhukov, V.A. Pea marker data-base (PMD)—A new online database combining known pea (*Pisum sativum* L.) gene-based markers. *PLoS ONE* **2017**, *12*, e018713.
